# *Claroideoglomus etunicatum* affects the diversity and composition of the rhizosphere microbial community to help tall fescue resist saline–alkali stress

**DOI:** 10.3389/fmicb.2025.1749714

**Published:** 2026-01-23

**Authors:** Hui Liu, Xiliang Song, Peiliang Zhang, Lu Liu, Chunhua Li

**Affiliations:** College of Life Sciences, Dezhou University, Dezhou, China

**Keywords:** arbuscular mycorrhizal fungi (AMF), biomass, soil microbial composition, soil microbial diversity, soil saline–alkali

## Abstract

**Introduction:**

Arbuscular mycorrhizal fungi (AMF) and plant rhizosphere microbes reportedly enhance plant tolerance to abiotic stresses and promote plant growth in contaminated soils. Soil salinization represents a severe environmental problem. Although the influence of AMF in the phytoremediation of saline–alkali soils has been fully demonstrated, the underlying interactive mechanisms between AMF and rhizosphere microbes are still unclear.

**Methods:**

A greenhouse pot experiment was conducted to explore the effects of AMF (*Claroideoglomus etunicatum*) on tall fescue growth promotion and the rhizosphere microbial community in saline–alkali soils. We aimed to investigate the mechanism of AMF affecting plant growth under saline–alkali stress conditions via interactions with rhizosphere microbes.

**Results:**

We found that AMF significantly increased plant shoot, root, and total biomass in saline–alkali stress soil. AMF significantly increased the diversity of bacterial and fungal communities and altered their composition. For bacteria, the AMF inoculation treatment (M+) showed higher relative abundance of Proteobacteria, Actinobacteriota, and Firmicutes and lower relative abundance of Acidobacteriota and Chloroflexi compared to the no-AMF application treatment (M−). For fungi, the M+ treatment showed lower relative abundance of Ascomycota and higher relative abundance of Mortierellomycota compared to the M− treatment. Furthermore, structural equation modeling (SEM) revealed that AMF promoted plant growth under saline–alkali stress conditions mainly by regulating the diversity of bacterial communities in the rhizosphere soil.

**Discussion:**

This study provides a theoretical basis for improving plant adaptation to saline–alkali stress through soil microbial management practices.

## Introduction

Saline–alkali stress has become one of the most important abiotic stresses and a serious threat to plant growth and agricultural production (Liu et al., 2022a; Anil et al., 2024). It is a major global issue that leads to many ecological and environmental problems, such as soil erosion, land desertification, forest and grassland degradation, and biodiversity reduction (Sun et al., 2016; Zai et al., 2021). In China, saline–alkali land is widely distributed and is expanding at a rate of approximately 1% per year (Du and Hu, 2021; Liu and Wang, 2021; Yang et al., 2022). Therefore, addressing the problem of saline–alkali land has attracted great public attention worldwide. In recent years, research has indicated that the use of biological methods to manage soil salinization is effective, eco-friendly, and sustainable, providing a breakthrough method for saline–alkali land management. In particular, plant species commonly form a symbiotic relationship with fungi, such as arbuscular mycorrhizal fungi (AMF), which may change their responses to saline–alkali stress.

AMF are a key group of soil microorganisms. They colonize plant roots to form mycorrhizal symbionts, completing their life cycle using photosynthesis-derived carbon provided by the plant, whereas the host plant receives water and mineral nutrients absorbed and transported by AMF in a mutually beneficial symbiosis (Chen et al., 2023). Several studies have revealed that symbiosis between AMF and plants under saline–alkali stress can promote plant growth and improve plant tolerance to saline–alkali conditions (Dastogeer et al., 2020; Qiu et al., 2020; Liang et al., 2021; Gao et al., 2023). Jia et al. (2019) found that under salt stress, AMF (*Rhizophagus irregularis*) increased plant (*Elaeagnus angustifolia*) growth, soluble sugar content, and soluble protein content. Furthermore, AMF enhanced secondary metabolism, promoted Ca^2+^ signal transduction, and improved reactive oxygen species (ROS) scavenging capacity. Liu et al. (2022b) reported that in tall fescue, AMF (*Claroideoglomus etunicatum*) under saline–alkali stress promoted plant biomass and nutrient uptake and regulated K^+^ and Na^+^ concentrations. Chen et al. (2023) demonstrated that AMF inoculation under salt stress alleviated toxic symptoms in *Alhagi sparsifolia* by promoting root growth, enhancing nutrient uptake, activating antioxidant enzyme activity, and regulating hormonal levels. Zhang D. J. et al. (2024) revealed that AMF (*Paraglomus occultum*) increased plant (*Gossypium hirsutum*) resistance to saline–alkali environments by promoting growth and regulating plant physiology. Therefore, the symbiosis between plants and AMF has great potential for improving saline–alkali tolerance and restoring saline–alkali lands, making it a major focus of research worldwide.

The structure of the rhizosphere microbial community changes with the external environment and is involved in improving plant saline–alkali tolerance (Zai et al., 2021; Mao et al., 2023; Ren et al., 2023; Zhou et al., 2023). Saline–alkali contamination in soils can inhibit the activity of microorganisms and disrupt physiological functions (Lee et al., 2022; Li et al., 2024; Zhang Y. et al., 2024). The *α*-diversity and network complexity of bacterial and fungal communities can be significantly reduced by high concentrations of saline–alkali stress (Yang et al., 2022; Tang et al., 2024). Some microbes can be enriched in the rhizosphere and are beneficial for plant growth under saline–alkali stress (Liu et al., 2021; Liu et al., 2023a; Ren et al., 2023; Tang et al., 2023). Rhizosphere bacteria (e.g., *Bacillus*, *Pseudomonas*, *Micrococcus*, *Arthrobacter*, *Burkholderia*, and *Paenibacillus*) can promote plant growth in saline–alkali-contaminated soils by increasing the N:P ratio, K:Na ratio, proline content, and superoxide dismutase activity, as well as slow the decline of soil nutrient levels (Liu et al., 2021; Duan et al., 2023; Wang et al., 2023). Some fungi, such as *Penicillium*, *Trichoderma*, and *Apophysomyces*, are highly tolerant to saline–alkali stress, and their presence can enhance plant growth by improving the K^+^/Na^+^ ratio, stimulating the antioxidant system, and promoting photosynthesis (Zai et al., 2021; Jin et al., 2022; Liu et al., 2023b). However, whether AMF affect the rhizosphere microbial community and whether AMF withstand saline–alkali stress by regulating the rhizosphere microbial community are unclear. In this study, high-throughput DNA sequencing and statistical analysis were used to investigate the diversity of the rhizosphere microbial community of the tall fescue (*Festuca elata* “Crossfire II”) and its correlation with the application of AMF in the plant’s adaptation to saline–alkali stress. This study aimed to explore the effects of saline–alkali stress on rhizosphere soil microorganisms and to investigate how AMF inoculation promotes plant growth and tolerance to saline–alkali stress by regulating the diversity and structure of the rhizosphere soil microbial community.

## Materials and methods

### Plant material and arbuscular mycorrhizal fungi

Seeds of tall fescue (*Festuca elata* “Crossfire II”) were supplied by Clover Group Co., Ltd., Beijing, China. The seeds were surface sterilized with 70% alcohol solutions for 2 min and 1% sodium hypochlorite for 5 min, followed by five washes with sterilized water (shaking for 5 min each time) (Ding et al., 2016). The sterilized seeds were then sown in pots (21 cm diameter × 16 cm height) containing 1.2 kg of soil. Soil was collected from the agricultural garden of Dezhou University, China. After the removal of roots and gravel, the soil was air-dried, ground, and passed through a 2-mm nylon sieve. The soil had a pH of 7.54. The soil contained 5.70 g/kg organic carbon, 0.60 g/kg total nitrogen, 0.62 g/kg total phosphorus, and 11.20 mg/kg available phosphorus. A total of 20 seeds were sown per pot. After germination, seedlings were thinned to 15 uniform plants per pot. The pots were then randomly placed in a greenhouse located at Dezhou University under conditions of 20–28 °C, 50–60% relative humidity, and natural daylight.

The AMF used was *C. etunicatum*, which was propagated in a sterilized sand–zeolite mixture using *Sorghum bicolor* as a host under potted conditions. When growth was completed (three-month cycle), the pots were air-dried, and the potting mixture—consisting of spores, colonized root fragments, and hyphae—was used as AMF inoculum. The spore density of AMF, counted under a dissecting microscope, was ca. Three hundred spores per 100 g of inoculum, which were isolated and collected using the wet sieving and decantation method and sucrose centrifugation.

### Experimental design

The experiment consisted of a completely randomized design with two factors. The first factor, saline–alkali treatment, included four intensities: 0, 200, 400, and 600 mmol/L. A total of four salts—NaCl, Na_2_SO_4_, NaHCO_3_, and Na_2_CO_3_—were mixed in a 9:1:1:9 M ratio to simulate a range of mixed saline–alkali stress conditions, according to the ion composition of salt–alkali soils in the northwest region of Shandong Province. The saline–alkali levels used in the experiment reflected the range of natural environmental conditions and did not cause extreme plant mortality, except for the 600 mM saline–alkali stress treatment. Furthermore, 300 mL of each saline–alkali solution was applied gradually to each pot to avoid serious osmotic shock by successively adding 100 mL every two days; an equal volume of distilled water was poured into the control pots. Soil moisture content was monitored daily using a soil moisture probe (ECH_2_O Check; Decagon Devices, Pullman, WA, United States), and any lost moisture was replenished with distilled water. The second factor, AMF inoculation treatment, included two levels: inoculation with *C. etunicatum* (M+; 100 g of AMF inoculum was added to each pot) and no AMF application (M−; 100 g of sterilized inoculum and 50 mL of non-sterilized inoculum filtrate, passed through a 10 μm sieve, to maintain similar microbial communities free of AMF propagules). The combination of these two factors resulted in a total of eight treatments (4 saline–alkali stress levels × 2 AMF inoculation status). Each treatment had five replicates, resulting in a total of 40 plastic pots.

### Sample collection and index determination

Plant and soil samples were collected from the pots 90 days after the start of the experiment. The plant samples were separated into shoot and root portions and were dried at 105 °C for 30 min to kill the tissue and inactivate enzymes, followed by drying at 75 °C to a constant weight to measure dry weight (Li et al., 2016; Ci et al., 2021; Xu et al., 2025). The plant roots (ca. 2 g) were randomly sampled and cut into 1-cm-long fragments. The fragments were cleared with 10% (w/v) KOH and stained with 0.05% (w/v) trypan blue in lactophenol (Phillips and Hayman, 1970). Mycorrhizal colonization was quantified as the percentage of AMF-colonized root length compared to the observed root length.

To analyze the composition of bacterial and fungal communities in the rhizosphere soil samples under different saline–alkali stress treatments, microbial profiling was performed by Majorbio Bio-Pharm Technology Co., Ltd. (Shanghai, China). Microbial DNA from each sample was extracted using the E.Z.N.A.^®^ Soil DNA Kit (Omega Bio-tek, Norcross, GA, United States). Bacterial 16S rRNA genes were amplified using the primers 338F (5′-ACTCCTACGGGAGGCAGCAG-3′) and 806R (5′-GGACTACHVGGGTWTCTAAT-3′). Fungal ITS genes were amplified using the primers ITS1F (5′-CTTGGTCATTTAGAGGAAGTAA-3′) and ITS2R (5′-GCTGCGTTCTTCATCGATGC-3′).

### Statistical analysis

Plant and soil data were statistically analyzed using two-way analysis of variance to evaluate the main effects (Saline–alkali, S; AMF inoculation, M) and their interactions using SPSS 27.0 (IBM), and means were separated using Duncan’s test at a *p*-value of <0.05. Non-metric multidimensional scaling (NMDS) analysis based on the binary_sorensen_dice distance was performed to assess differences in microbial community composition among the treatments. To estimate the direct and indirect effects of saline–alkali stress and AMF inoculation on tall fescue biomass, Structural equation modeling (SEMs) were fitted to our data using SPSS Amos v. 29.0. We used bacterial diversity (BD) and fungal diversity (FD) as explanatory variables.

## Results

### AMF colonization rate and plant biomass allocation

**Figure 1:**
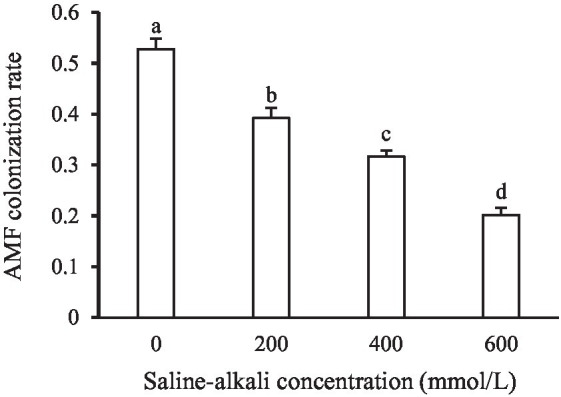
Colonization rate of arbuscular mycorrhizal fungi (AMF) under saline–alkali stress conditions at the end of the experiment. Values are means ± standard error (SE). Different letters denote means that are significantly different (*p* < 0.05).

The average AMF colonization rate ranged from 20.2 to 52.8% across the four saline–alkali treatments (Figure 1). The AMF colonization rate was significantly affected by saline–alkali stress (Table 1), and the AMF colonization rate decreased with increasing saline–alkali stress concentration (Figure 1).

**Table 1:** One-way ANOVA showing the effects of saline–alkali stress (S) on the arbuscular mycorrhizal fungi (AMF) colonization rate in tall fescue roots, and two-way ANOVA showing the effects of arbuscular mycorrhizal fungi (M) and saline–alkali stress (S) on the shoot biomass, root biomass, total biomass, and the root:shoot ratio of tall fescue.

Index	AMF colonization rate		Shoot biomass		Root biomass		Total biomass		Root:shoot ratio	
	*F*	*P*	*F*	*P*	*F*	*P*	*F*	*P*	*F*	*P*
Arbuscular mycorrhizal fungi (M)			27.503	**<0.001**	16.639	**<0.001**	27.412	**<0.001**	2.887	0.099
Saline–alkali stress (S)	65.784	**<0.001**	59.245	**<0.001**	58.136	**<0.001**	63.287	**<0.001**	10.487	**<0.001**
M × S			3.067	**0.042**	3.808	**0.019**	3.120	**0.040**	3.749	**0.021**

Bold numbers indicate significant differences at the 0.05 level.

**Figure 2:**
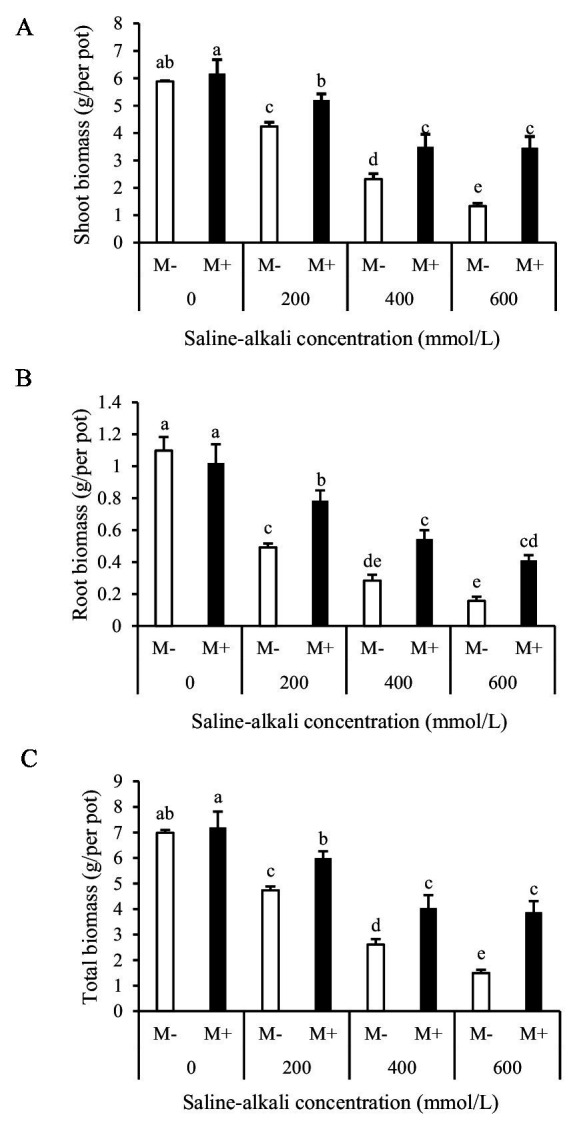
Shoot **(A)**, root **(B)**, and total biomass **(C)** of tall fescue with (M+) and without (M−) arbuscular mycorrhizal fungi under saline–alkali stress. Values are means ± standard error (SE). Different letters denote means that are significantly different (*p* < 0.05).

There was a significant interactive effect of AMF and saline–alkali stress on shoot, root, and total biomass (Table 1). The M+ plants had 23–160% higher shoot biomass (Figure 2A), 59–163% higher root biomass (Figure 2B), and 27–160% higher total biomass (Figure 2C) compared to the M− plants under the 200, 400, and 600 mmol/L saline–alkali treatments. No remarkable differences in shoot, root, and total biomass were detected between the M+ and M− plants under the 0 mmol/L (non-stress) saline–alkali treatment (Figure 2).

**Figure 3:**
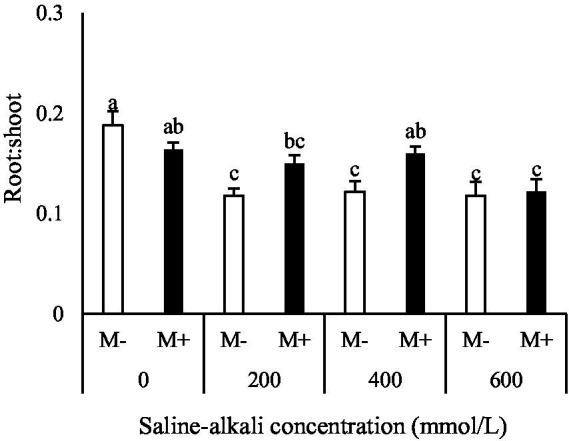
Root:shoot ratio of tall fescue with (M+) and without (M−) arbuscular mycorrhizal fungi under saline–alkali stress. Values are means ± standard error (SE). Different letters denote means that are significantly different (*p* < 0.05).

The root:shoot ratio is an important indicator of plants’ allocation of resources. The root:shoot ratio was significantly higher in the M+ plants than in the M− plants at relatively low saline–alkali stress levels (200 and 400 mmol/L) (Table 1 and Figure 3). This indicated that, with the assistance of AMF, tall fescue allocated more resources to the growth and development of below-ground parts. However, no significant differences were observed between the M+ and M− plants under non-stress (0 mmol/L) or high-stress (600 mmol/L) conditions (Figure 3).

### Rhizosphere soil microbial diversity and richness

The AMF × saline–alkali stress interaction significantly affected the bacterial Shannon index and Simpson index (Table 2). The M+ treatment showed a higher Shannon index (5–19%) and a lower Simpson index (40–94%) compared to the M− treatment under the 0, 200, 400, and 600 mmol/L saline–alkali conditions (Table 3). The richness indices (Ace and Chao) of bacteria were also increased by AMF (9 and 11% on average) (Table 3).

**Table 2:** Two-way ANOVA showing the effects of arbuscular mycorrhizal fungi (AMF) and saline–alkali stress (S) on the diversity indices (Shannon and Simpson) and richness indices (Ace and Chao) of bacteria and fungi in tall fescue rhizosphere soil.

Index	Shannon				Simpson				Ace				Chao			
	Bacteria		Fungi		Bacteria		Fungi		Bacteria		Fungi		Bacteria		Fungi		*F*	*P*	*F*	*P*	*F*	*P*	*F*	*P*	*F*	*P*	*F*	*P*	*F*	*P*	*F*	*P*
Arbuscular mycorrhizal fungi (M)	83.066	**<0.001**	24.077	**<0.001**	174.975	**<0.001**	47.780	**<0.001**	5.794	**0.022**	22.127	**<0.001**	29.544	**<0.001**	14.078	**<0.001**
Saline–alkali stress (S)	22.943	**<0.001**	7.711	**<0.001**	16.320	**<0.001**	5.783	**0.003**	1.024	0.395	9.561	**<0.001**	3.268	**0.034**	4.024	**0.015**
M × S	2.944	**0.048**	3.008	**0.045**	17.216	**<0.001**	0.610	0.614	0.109	0.954	0.306	0.821	0.070	0.975	0.014	0.998

Bold numbers indicate significant differences at the 0.05 level.

**Table 3:** Diversity and richness indices of bacteria and fungi in the rhizosphere soil of tall fescue with (M+) and without (M−) arbuscular mycorrhizal fungi under saline–alkali stress.

Index		Shannon	Simpson	Ace	Chao
Bacteria					
0 mmol/L	M−	6.48 ± 0.06b	0.005 ± 0.001c	4756.22 ± 345.70ab	4597.15 ± 146.02bc
	M+	7.05 ± 0.05a	0.003 ± 0.000d	5118.60 ± 85.44a	5023.98 ± 169.86a
200 mmol/L	M−	6.64 ± 0.07b	0.014 ± 0.004b	4647.39 ± 185.83ab	4432.43 ± 135.14 cd
	M+	7.05 ± 0.04a	0.003 ± 0.000d	4957.00 ± 146.49ab	4936.11 ± 61.75ab
400 mmol/L	M−	6.66 ± 0.05b	0.018 ± 0.002b	4566.18 ± 100.34ab	4345.91 ± 116.34 cd
	M+	6.99 ± 0.09a	0.004 ± 0.000 cd	4909.43 ± 194.26ab	4838.19 ± 122.16ab
600 mmol/L	M−	5.88 ± 0.13c	0.048 ± 0.002a	4268.46 ± 403.55b	4151.41 ± 119.42d
	M+	6.65 ± 0.11b	0.003 ± 0.000 cd	4816.34 ± 168.08ab	4693.40 ± 125.29abc
Fungi					
0 mmol/L	M−	4.26 ± 0.13a	0.049 ± 0.007bc	475.39 ± 13.54bc	478.66 ± 21.62ab
	M+	4.28 ± 0.08a	0.031 ± 0.003d	539.10 ± 16.77a	547.07 ± 19.91a
200 mmol/L	M−	3.91 ± 0.07bc	0.060 ± 0.008ab	420.99 ± 17.54de	428.26 ± 17.97bc
	M+	4.25 ± 0.02a	0.033 ± 0.003 cd	492.47 ± 16.56ab	493.25 ± 17.29ab
400 mmol/L	M−	3.80 ± 0.07 cd	0.071 ± 0.006a	427.48 ± 16.79cde	429.25 ± 30.04bc
	M+	4.13 ± 0.06ab	0.040 ± 0.008 cd	471.93 ± 15.52bcd	487.81 ± 31.21ab
600 mmol/L	M−	3.58 ± 0.13d	0.076 ± 0.005a	394.47 ± 20.07e	397.99 ± 32.66c
	M+	4.14 ± 0.11ab	0.045 ± 0.002bcd	440.64 ± 18.23bcde	463.01 ± 16.17bc

Values are means ± standard error (SE). Different letters denote means that are significantly different (*p* < 0.05).

For fungi, the Shannon index was significantly affected by the interaction between AMF inoculation and the saline–alkali stress treatment (Table 2). The Shannon index was significantly higher in the M+ treatment than in the M− treatment under the 200, 400, and 600 mmol/L saline–alkali conditions; no significant difference in the Shannon index was detected between the M+ and M− treatments under the 0 mmol/L (non-stress) saline–alkali condition (Table 3). The Simpson index was significantly increased by the saline–alkali treatment (25% on average) but decreased by AMF inoculation (42% on average). In contrast, bacterial richness indices (Ace and Chao) were significantly affected by both AMF and saline–alkali stress but not by their interactions (Table 3). Saline–alkali stress significantly decreased bacterial richness indices (Ace and Chao). Ace and Chao indices in the M+ treatment were 13 and 15% higher, respectively, than in the M− treatment, regardless of saline–alkali stress (Table 3).

### Rhizosphere soil microbial community composition

**Figure 4:**
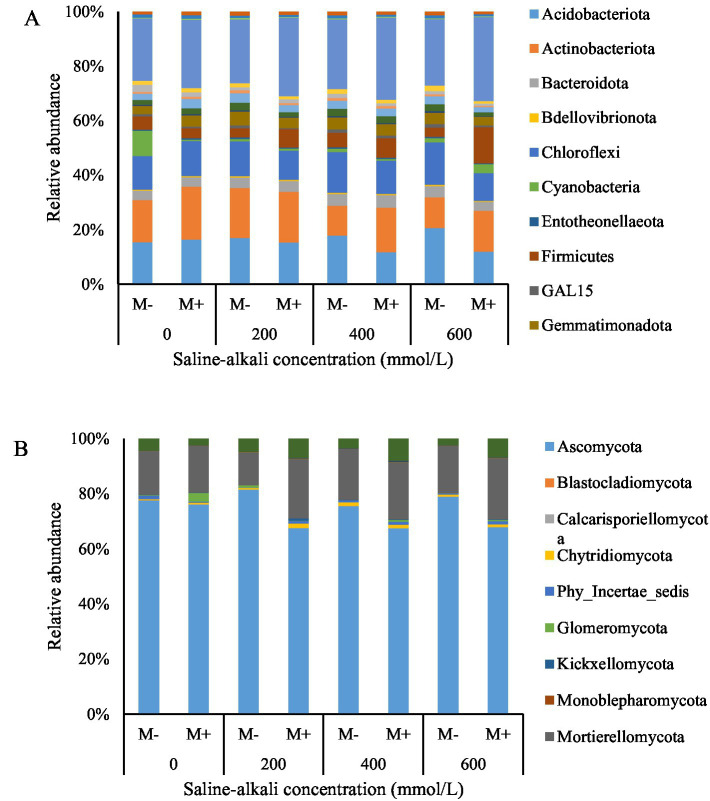
Relative abundance of bacterial **(A)** and fungal **(B)** phyla in the rhizosphere soil of tall fescue with (M+) and without (M−) arbuscular mycorrhizal fungi under saline–alkali stress. Data are presented as mean values from five rhizosphere soil samples of tall fescue.

The most abundant bacterial phyla were Proteobacteria, Acidobacteriota, Actinobacteriota, Chloroflexi, and Firmicutes, whose relative abundance together accounted for 71–82% of the total bacterial community (Figure 4A). AMF significantly decreased the relative abundance of Acidobacteriota and Chloroflexi but increased the relative abundance of Proteobacteria, Actinobacteriota, and Firmicutes under the 200, 400 and 600 mmol/L saline–alkali treatments (Figure 4A). The most abundant fungal phyla were Ascomycota, Mortierellomycota, and unclassified fungi, and their relative abundance together accounted for 96–99% of the overall fungal community (Figure 4B). AMF increased the relative abundance of Mortierellomycota and unclassified fungi in the rhizosphere soil while concurrently reducing the relative abundance of Ascomycota, regardless of whether saline–alkali stress was low or high. However, no significant difference in these fungal groups was detected between the M+ and M− treatments under the 0 mmol/L (non-stress) saline–alkali condition (Figure 4B).

**Figure 5:**
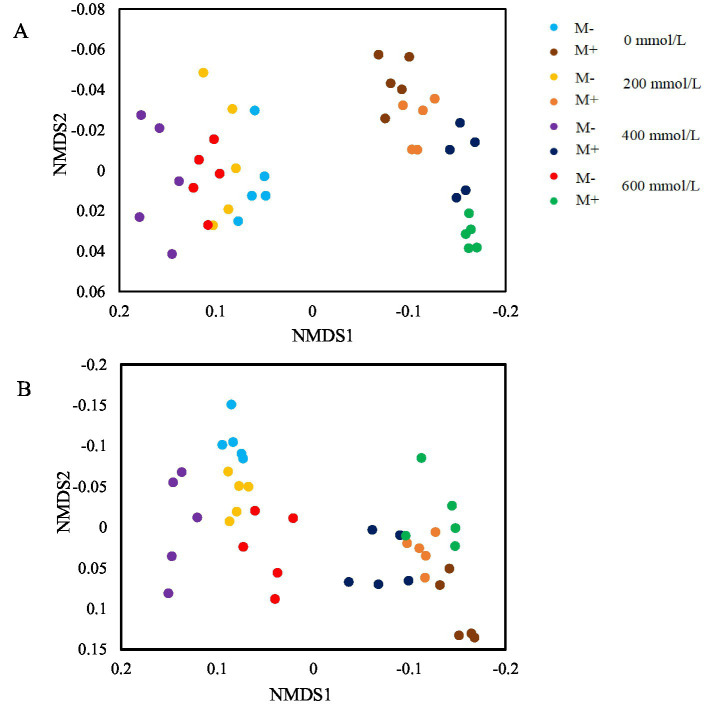
Non-metric multidimensional scaling (NMDS) ordination of bacterial **(A)** and fungal **(B)** community composition in the rhizosphere soil of tall fescue under saline–alkali stress conditions. Data are presented as mean values from five rhizosphere soil samples of tall fescue.

A non-metric multidimensional scaling (NMDS) analysis of microbial community composition was performed based on 16S rRNA and ITS gene sequences from rhizosphere soil using the binary_sorensen_dice. The NMDS analysis showed that bacterial (ANOSIM: *R* = 0.4048, *p* = 0.001, Figure 5A) and fungal (ANOSIM: *R* = 0.1586, *p* = 0.008, Figure 5B) community compositions in the rhizosphere soil of tall fescue under AMF colonization were distinct from those without AMF colonization under both non-stress (0 mmol/L) and saline–alkali stress (200, 400, and 600 mmol/L) conditions, indicating a significant effect of AMF colonization on soil microbial community structure.

### Direct and indirect effects of AMF and saline–alkali stress on the growth of tall fescue

**Figure 6:**
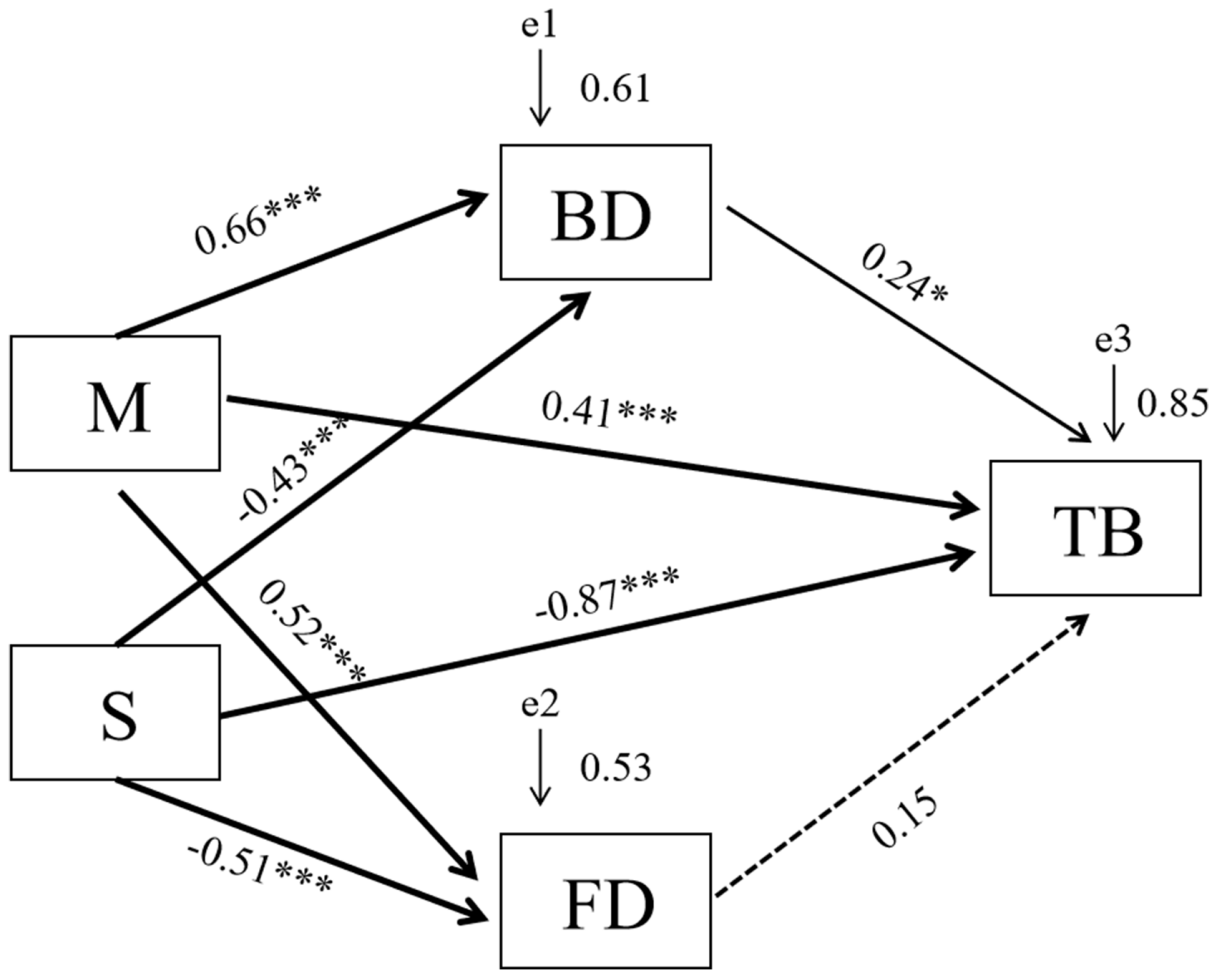
Structural equation model showing the hypothesized causal relationships between bacterial and fungal diversity and tall fescue total biomass under saline–alkali stress conditions. Solid lines and dashed lines represent significant and non-significant pathways, respectively (^***^p < 0.001, ^**^p < 0.01, and ^*^p < 0.05). M, AMF inoculation; S, saline–alkali stress; BD, bacterial diversity; FD, fungal diversity; TB, total biomass.

The direct and indirect effects of AMF and saline–alkali stress on the growth (total biomass) of tall fescue were analyzed using structural equation modeling (SEM). AMF and saline–alkali stress had direct effects on total biomass, as well as indirect effects mediated through measured intermediate parameters: AMF inoculation increased the total biomass of tall fescue by indirectly increasing bacterial diversity, but not fungal diversity (Figure 6).

## Discussion

Saline–alkali stress is one of the most important abiotic stresses that severely threatens plant growth and influences the productivity of agriculture (Chen et al., 2019; Zhang et al., 2019; Liu et al., 2023b). Previous studies have mostly compared differences in plant growth between treatments with and without saline–alkali stress, with little attention paid to the effects of AMF on plant growth across different levels of saline–alkali stress. In the present study, regardless of AMF inoculation, the biomass of tall fescue decreased significantly as saline–alkali concentrations continued to increase, which may be due to the negative effects of high saline–alkali concentrations on AMF colonization rates. However, AMF inoculation significantly improved plant growth parameters, with higher shoot, root, and total biomass in the M+ plants than in the M− plants, and the beneficial effect of AMF became more obvious with increasing saline–alkali concentration. Mycorrhizal plants may attenuate the toxic effects of saline–alkali stress by maintaining higher biomass (Parvin et al., 2020; Zhang et al., 2023), thereby playing a significant role under adverse conditions. Furthermore, the M+ plants showed a higher root:shoot ratio compared to the M− plants at relatively low saline–alkali stress levels (200 and 400 mmol/L), which could increase the root surface area for efficient water uptake and help plants survive water shortage (Chen et al., 2023). Similar results were found by Gao et al. (2023) and Zhang D. J. et al. (2024). The decrease in the M+ plants’ biomass was smaller than that in the control treatment, which facilitated better adaptation of tall fescue to saline–alkali stress.

Related studies have shown that changes in soil microbial diversity, structure, and function affect the ability of plants to withstand stress (Hao et al., 2022; Liu B. S. et al., 2022). AMF have the potential to alter plant root exudates and produce new biochemicals to reshape microbial community composition in the rhizosphere (Xing et al., 2024). In this study, we found that AMF inoculation alleviated the negative effect of saline–alkali stress on bacterial and fungal diversity in the rhizosphere soil of tall fescue, with higher bacterial and fungal diversity observed in the M+ plants than in the M− plants under 200, 400, and 600 mmol/L saline–alkali stress conditions. In addition, AMF inoculation altered the abundance of several bacterial and fungal groups, including increasing the relative abundance of Proteobacteria, Actinobacteriota, Firmicutes, and Mortierellomycota. Proteobacteria are among the most abundant bacterial groups in terrestrial soils (Wang et al., 2021) and can survive under extreme conditions (Liao et al., 2023). They participate in soil nitrification and oxidation processes, thereby increasing soil nutrients. Actinobacteriota generally control the utilization of sugar in the soil, and Firmicutes are concentrated in contaminated soils and are effective in bioremediation and stress tolerance (Ci et al., 2021; Wang et al., 2024). Most species of Mortierellomycota are saprophytic and primarily participate in the decomposition of soil organic carbon (Liu B. S. et al., 2022; Bai et al., 2024). These results suggest that AMF may enhance resistance to saline–alkali stress by regulating the diversity and composition of the rhizosphere microbial community, and AMF may attract some bacteria and fungi in the tall fescue rhizosphere soil, which is useful for plant growth.

Our results showed that saline–alkali stress and AMF inoculation affected both the diversity and composition of bacterial and fungal communities in the tall fescue rhizosphere. An important question is, “Which bacteria or fungi contribute most to the differences in tall fescue growth between M+ and M− treatments under saline–alkali stress conditions?” In our study, SEM was performed to examine the relationships between microbial community and tall fescue total biomass under saline–alkali stress conditions. The results showed that AMF inoculation directly increased tall fescue total biomass and indirectly promoted growth by increasing bacterial community diversity and regulating bacterial community composition, compared to fungal communities, under saline–alkali stress conditions. Similar results have been reported by Lu et al. (2023), who demonstrated that AMF symbiosis recruited rhizosphere bacterial communities to improve soil phosphate mobilization and regulate sulfur uptake, and by Kavadia et al. (2021), who found that inoculation with AMF and nitrogen-fixing bacteria (*Sinorhizobium meliloti*) increased shoot biomass and nitrogen accumulation. Combined with the above results, our study suggests that AMF may alleviate the negative effects of saline–alkali stress on plants and microbes by enhancing the synergistic interaction among microbial communities, providing a theoretical basis for improving plant adaptation to saline–alkali stress through soil microbial management.

## Conclusion

AMF inoculation significantly alleviated the damage caused by different concentrations of saline–alkali stress. The effect became more pronounced as the concentration of saline–alkali increased. Our study provides evidence that AMF enhances plant growth and resistance to saline–alkali stress by regulating rhizosphere soil microbial communities, thereby improving our understanding of the underlying mechanisms.

## Data Availability

The original contributions presented in the study are publicly available. This data can be found here: [PRJNA1394446].
